# Case report: Mixed dementia associated with autoantibodies targeting the vesicular glutamate transporter 2

**DOI:** 10.3389/fpsyt.2023.1227824

**Published:** 2023-07-12

**Authors:** Niels Hansen, Bianca Teegen, Sina Hirschel, Jens Wiltfang, Björn H. Schott, Claudia Bartels, Caroline Bouter

**Affiliations:** ^1^Department of Psychiatry and Psychotherapy, University Medical Center Göttingen, Göttingen, Germany; ^2^Clinical Immunological Laboratory Prof. Stöcker, Groß Grönau, Germany; ^3^German Center for Neurodegenerative Diseases (DZNE), Göttingen, Germany; ^4^Neurosciences and Signaling Group, Institute of Biomedicine (iBiMED), Department of Medical Sciences, University of Aveiro, Aveiro, Portugal; ^5^Leibniz Institute for Neurobiology, University of Magdeburg, Magdeburg, Germany; ^6^Department of Nuclear Medicine, University Medical Center Göttingen, Göttingen, Germany

**Keywords:** autoimmunity, cognition, VGlut2, autoantibodies, Alzheimer's dementia, depression

## Abstract

**Background:**

Autoantibodies against the vesicular glutamate transporter type 2 (VGlut2) can trigger impaired synaptic signaling and are described here for the first time in association with mixed dementia.

**Methods:**

We report on a 71-year-old female patient with a dementing syndrome who underwent a thorough dementia diagnosis including neuropsychological testing, magnetic resonance imaging (MRI), ^18^F-fluorodesoxyglucose positron emission tomography (FDG-PET), and a spinal tap to search for neural autoantibodies.

**Results:**

Our patient exhibited mixed dementia. Her CSF revealed elevated ptau 181 protein and a reduced Aß42/40 ratio indicating Alzheimer's disease (AD) pathology. In addition, neuropsychological testing showed a profile consistent with AD with impaired memory, reduced semantic word fluency, naming disorder, and impaired visuoconstructive skills. Nevertheless, in-depth neuropsychological testing also revealed marked psychomotor slowing and visuospatial perceptual impairments that are more indicative of the presence of DLB. Overall, her dementia is more likely of mixed pathology. In addition, we repeatedly detected VGlut2 autoantibodies in her serum.

**Conclusion:**

To the best of our knowledge, this report is the first to describe mixed dementia associated with VGlut2 autoantibodies.

## 1. Introduction

An undisturbed glutamatergic excitatory neurotransmission between synapses can only occur when glutamate is transported *via* vesicles such as the vesicular glutamate transporter 2 (VGlut2). Several lines of evidence (1, 9) support the notion that Alzheimer's disease (AD) can be understood as a synaptopathy. The vesicular glutamate transporter 2 (VGlut2) as part of the glutamatergic systems is involved in AD. This assumption is based on observations that VGlut2 expression in the dorsolateral prefrontal cortex is reduced in Alzheimer's disease patients (2) as well as the fact that increased amyloid beta peptide accumulation takes place in the VGlut2 terminals (3). Antibodies against the VGlut2 transporter could cause synaptic dysfunction. In our case report, we describe the diagnosis of clinically and cerebrospinal fluid (CSF)-based AD associated with VGlut2 autoantibodies, which has never been described to our knowledge. The role of VGlut2 autoantibodies in AD is unknown, but they may be associated with synaptic dysfunction, supporting the hypothesis of AD as synaptopathy. Through this report, the potentially diverse spectrum of neuronal autoantibodies in cognitive disorders (4, 5) can be supported.

## 2. Case report

**Figure 1:**
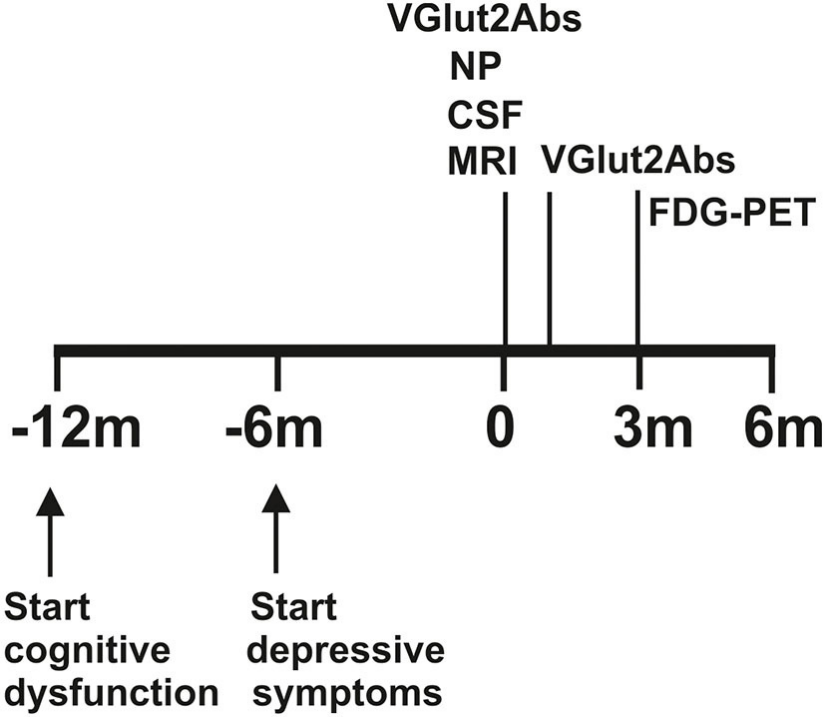
Time course of symptoms and investigations. MRI, magnetic resonance imaging; CSF, cerebrospinal fluid; FDGPET, fluorodesoxyglucose positron emission computed tomography; M, month; MRI, magnetic resonance imaging; NP, neuropsychological testing; VGlut2 abs, vesicular glutamate transporter 2 autoantibodies.

**Figure 2:**
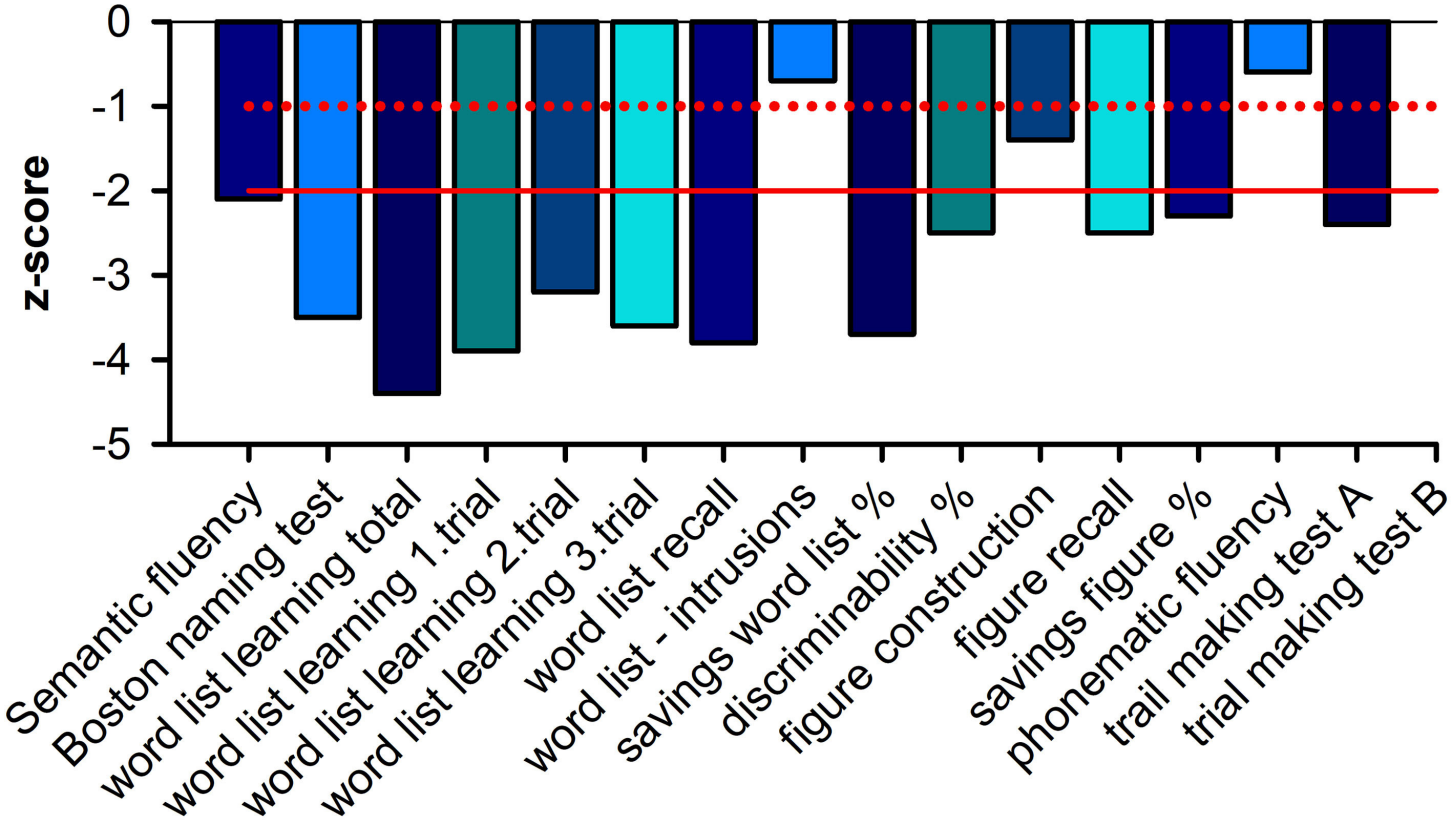
Neuropsychological profile.

**Figure 3:**
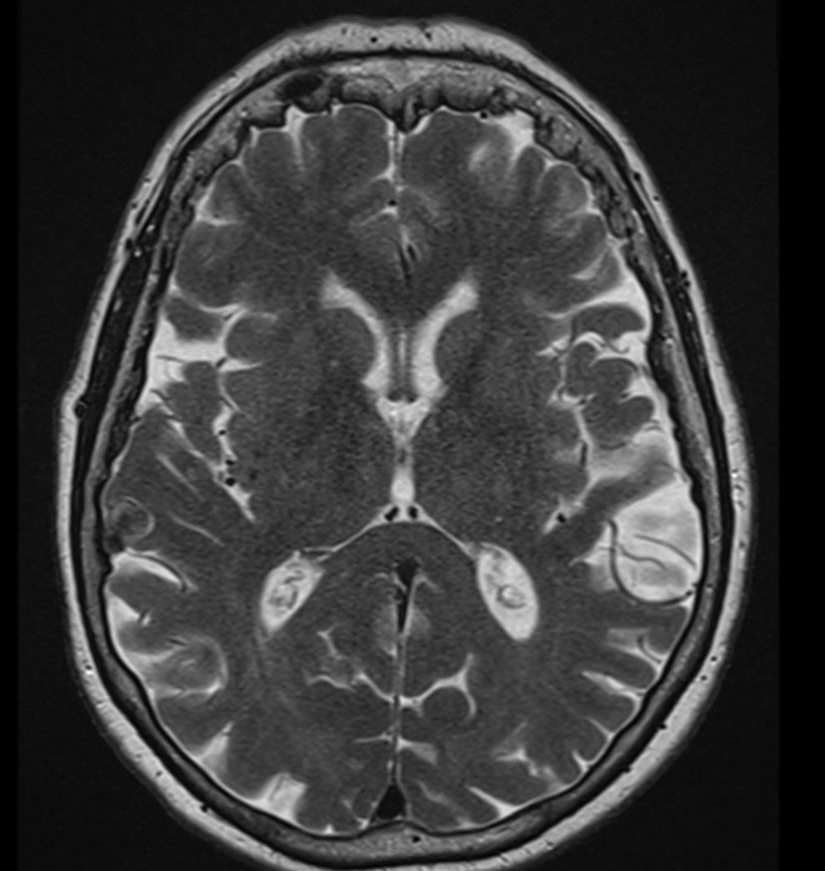
Magnetic resonance imaging. Magnetic resonance imaging (MRI) of the brain shows minor cerebral microangiopathy with periventricular vascular lesions in the white matter.

**Figure 4:**
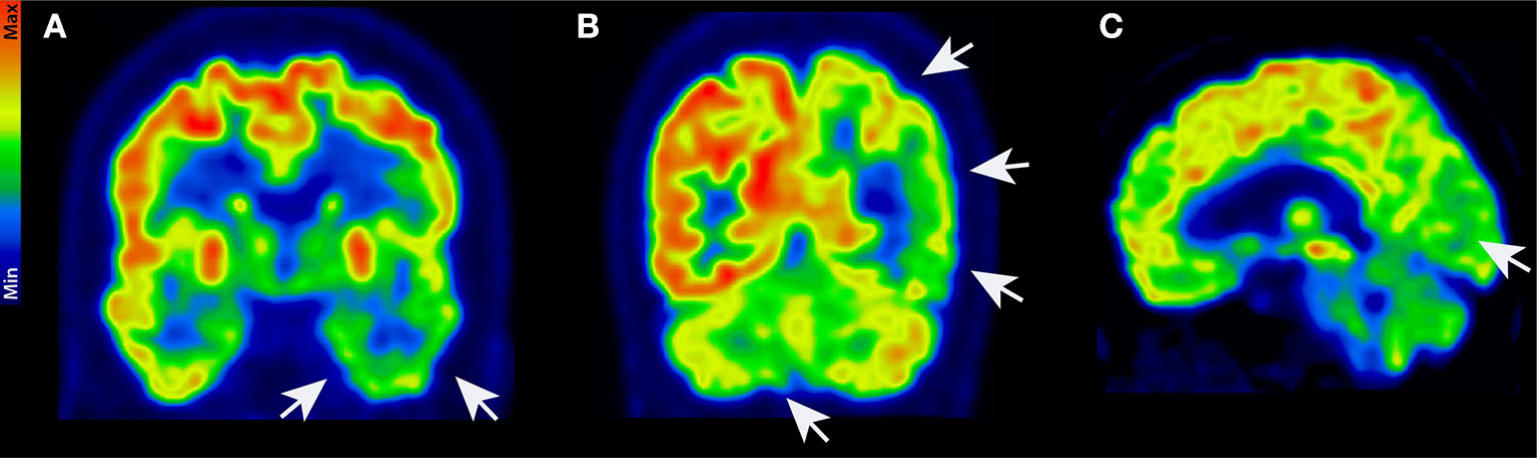
^18^F-FDG-PET. ^18^F-FDG-PET in coronal **(A, B)** and sagittal **(C)** view with distinct hypometabolism in the left temporal **(A)**, left parietal, and right cerebellar cortex **(B)**. Furthermore, hypometabolism in the left primary visual cortex was detected **(C)**. White arrows show brain areas with lower tracer uptake.

A 71-year-old female patient presented to the emergency ward in our psychiatric clinic. For about half a year, she had been suffering from a depressed mood, pronounced restlessness, a concentration disorder, diffuse anxiety, and sleep disturbances (Figure 1). The depressive symptoms had been very obvious for 3 weeks. She had no appetite and cried more often during conversations. Her sense of joy was reduced, and there was general listlessness. She suffers from early waking, feeling depressed in the morning, and feeling a deep sense of shame. She also described passive death wishes, but no concrete suicidal thoughts. She reports no relevant psychosocial stress factors and even when asked, describes no stress factors. After careful questioning of her daughter, we learned that her retention and memory disorders had already existed for a year (Figure 1). The daughter also spoke of her considerably impaired daily living skills, stating that she could probably no longer manage alone at home and depended on her daughter for help with self-care. Nursing help would also be needed at home to ensure that her medications are taken. The patient is a Kazakhstan native who has lived in Germany for 30 years. She is a widow with two children. She is a trained hotel manager, a pensioner, and lives alone. Her daughter has the power of attorney for her healthcare. There are no known psychiatric diseases in her family history. She had no known somatic diseases. In her psychopathological examination, we observed indications of concentration and comprehension disturbances as well as impaired retentiveness and memory. Furthermore, she worries about the future. Her emotional state was depressed, and there was a disturbance of vital feelings. She also reported being less able to feel joy, a loss of interest, and reduced drive. She had also become socially withdrawn. Her neurological examination, however, was inconspicuous apart from the cognitive dysfunction. Her physical examination revealed no pathologies. Due to her serious memory problems that started before the depressive symptoms, we conducted extensive neuropsychological testing to make a differential diagnosis. The detailed neuropsychological examination revealed significant impairments in several cognitive domains such as orientation, semantic word fluency, confrontation naming, cognitive processing speed, visuomotor coordination, the clock test, action planning, working and figural memory, encoding and consolidation of verbal non-associated information, encoding and delayed recall of complex verbal content, visuoconstructive skills, and visual-spatial perception objectified (Figure 2, Table 1). However, her phonematic word fluency was not age appropriate. From a neuropsychological point of view, the aforementioned dysfunctions go beyond depression-related cognitive impairments and are compatible with dementia syndrome. Her major memory impairment, reduced semantic word fluency with naming disorders, and limited visuoconstructive skills indicate AD dementia. Atypical for AD dementia is the pronounced psychomotor slowing and visual-spatial perceptual disturbances. To enable additional differential diagnosis of neurodegenerative dementia, she underwent magnetic resonance imaging (MRI). Cranial MRI showed isolated cerebral lesions in the periventricular white matter (Figure 3). Cerebrospinal fluid was collected to ensure a differential diagnosis of inflammatory and neurodegenerative causes of dementia syndrome. Cerebrospinal fluid (CSF) revealed a constellation of biomarkers compatible with AD, e.g., a reduced Aß42 /40 ratio (0.05, reference level: >0.06) and elevated ptau 181 protein (70 pg/ml, reference level: <50 pg/ml). We detected VGlut2 autoantibodies (1:100) in serum samples, observed again in serum samples (1:1000) 1 month later. In addition, after incubating the patient's serum with rat and monkey brain tissues, immunohistochemistry revealed relevant spotty fluorescence of the granular layer in the hippocampus and streaky fluorescence in the thalamus and molecular layer in the cerebellum caused by the antibody against VGlut2. ^18^F-fluorodesoxyglucose positron emission tomography (^18^F-FDG-PET) of the brain showed left parietal and left temporal hypometabolism (Figure 4). In addition, a reduced tracer uptake was also detected in the right cerebellum consistent with diaschisis. Furthermore, the lower ^18^F-FDG uptake was detected in the left primary visual cortex (Figure 4). Overall, her PET provided evidence compatible with a DLB pattern or a pattern associated with a logopenic variant of primary progressive aphasia. To summarize her status, we suspect mixed dementia with Alzheimer's pathology and Lewy body pathology. The presence of AD is suggested by the CSF findings and her neuropsychological exam indicating impaired memory, reduced semantic word fluency and naming disorders, and impaired visuoconstructive skills. However, neuropsychological findings include marked psychomotor slowing and visuospatial perceptual disturbances, which are more indicative of the presence of DLB. However, note that biomarkers to diagnose DLB such as (123)-I-2-ß-carbomethoxy-3ß-(4-iodophenyl)-*N*-(3-fluoropropyl) nortropane single photon emission computed tomography (123I-FP-CIT SPECT) or [^123^I] metaiodobenzylguanidine (MIBG) cardiac scintigraphy were not explored. However, as she presented no other DLB symptoms other than her cognitive symptoms, i.e., evidence of Parkinson's, a REM sleep behavior disorder, or visual hallucinations, we did not investigate this potential component of Lewy pathology further. Overall, therefore, hers is probably a mixed pathology. As we consistently detected VGlut2 autoantibodies in her serum, that finding is relevant. We, therefore, diagnosed mixed dementia associated with VGlut2 autoantibodies. We ruled out an acute inflammatory CNS event because of her CSF results. To exclude a tumor, she also underwent whole-body FDG PET, which revealed a suspicious nodule near the anterior sternum. An additional dermatologic workup revealed nothing conclusive. Due to mixed dementia with components of Alzheimer's pathology, we started anti-dementia therapy with the acetylcholinesterase inhibitor donepezil at a 10 mg/d dosage. In the absence of evidence of inflammation in the CSF, we have refrained from immunotherapy with methylprednisolone off-label for the time being. We also had this patient undergo antidepressant effective therapy with sertraline 100 mg/day and quetiapine 150 mg/day. Quetiapine was discontinued during the course in favor of mirtazapine 45 mg/day after observing the lack of efficacy under mirtazapine. We find it quite tempting to postulate that her depressive symptoms are an expression of the underlying neurodegenerative mixed dementia associated with VGlut2 autoantibodies. Overall, her diagnosis is probably consistent with the prognosis of mild dementia syndrome due to AD, but it remains unclear whether the additional association with VGlut2 tends to favor the prognosis. After inpatient treatment, the patient experienced two more outpatient follow-up visits. Under antidepressant and anti-dementia therapy, her cognitive symptoms remained stable, and the depressive symptoms largely disappeared. She now presents a euthymic mood with no significant loss of drive and no dangerous behavior. Her medical therapy has been well tolerated, and no relevant side effects have occurred.

**Table 1:** Data of neuropsychological testing.

**Language**	**Raw value**	**Percentual rank**
**CERAD (age and education corrected standard)**		
Boston Naming Test	7	0
Semantic word fluency	7	2
Phonematic word fluency	8	27
**Attentional and executive functions**		
**WAIS-IV (age corrected standard)**		
Number Symbol Test	16	1
**TMT (age/education corrected standard)**		
Part A seconds	112	1
**Visuoconstruction**		
Clock test	3	na
CERAD		
Constructive practice: Signing off	8	8
**WAIS-IV (age corrected standard)**		
Mosaic test	16	5
**Learning and memory**		
**WAIS-IV (age corrected standard)**		
Number span forward	7	16
Number span backward	5	9
**CERAD (age and education corrected standard)**		
Learn word list: Sum Trial 1-3	7	0
Word list free retrieval	0	0
Savings word list (%)	0	0
Word List Recognition (%)	80	1
**WMS-IV (age corrected standard)**		
Logical memory I	9	0,1
Logical memory II	0	0,1
**CERAD (age and education corrected standard)**		
Constructive praxis: free recall	1	1
Saving figures (%)	13	1

CERAD, Consortium to Establish a Registry for Alzheimer's Disease; WAIS-IV, Wechsler Adult Intelligence Scale - Fourth Edition; WMS-IV, Wechsler Memory Scale - Fourth edition; TMT, Trial Making Test.

## 3. Discussion

Our report is the first to describe to the best of our knowledge an AD associated with the presence of VGlut2 autoantibodies in serum. Moreover, we provide a new perspective on a possible autoimmune involvement in AD, our data broadly support the hypothesis of AD synaptopathy by postulating a pathogenic role of VGlut2 autoantibodies resulting in synaptic dysfunction. However, note that this report only demonstrates an association between VGlut2 autoantibodies and AD but not their pathogenic role. On the other hand, one can argue that our patient presents no clear AD clinically and laboratory-diagnostically since marked psychomotor slowing and visual-spatial perceptual disturbances are both neuropsychologically evident. The pattern of cerebral hypometabolism also supports the diagnosis of alpha-synucleinopathy, so we suspect a mixed etiology of AD pathology and alpha-synucleinopathy, which has not previously been reported in association with VGlut2 autoantibodies. Interestingly, there is evidence that in alpha-synucleinopathy Parkinson's disease, VGlut2 receptor expression is upregulated in the dopaminergic neurons remaining after neurodegeneration (6). Assuming at least a co-pathology of alpha-synuclein in our patient, the presence of VGlut2 autoantibodies could thus be relevant if pathogenicity is postulated (by penetrating the blood–brain barrier). The cerebral cortex contributes to memory performance (7), supporting our hypothesis that by blocking VGlut2 function, autoantibodies may impair cognitive function and lead to dementia.

### 3.1. Limitations

To date, VGlut2 autoantibodies have not been reported in association with mixed dementia; therefore, this is an atypical clinical syndrome that should be interpreted with caution in a single case report.

### 3.2. Conclusion

Our case highlights the occurrence of VGlut2 antibodies in mixed dementia, supporting an additional dimension of neural autoantibody-associated dementia. The strength of the case report lies in the new observation of the occurrence of VGlut2 autoantibodies in dementia syndrome. Although the pathophysiological basis of the symptoms can be traced back to a disturbed glutamate vesicle transport and altered synaptic information transmission and encoding, such a pathophysiological basis as the cause of the symptoms remains speculative and cannot be proven at present. However, this report highlights a new rare autoantibody not previously described in the context of cognitive impairment that should be further investigated in larger cohorts, particularly in relation to AD pathology but also alpha-synucleinopathy. Recently, in a large study of 920 patients with neurodegenerative dementias, 0.8% were found to have neural autoantibodies (8). In this study, no abnormalities were found on MRI, and a small proportion was found with pleocytosis in CSF (8). This study confirms that autoantibody-associated predominantly neurodegenerative dementias that exist would benefit from a modified therapeutic approach. Such a therapeutic approach with immunotherapy and standard therapy should be investigated in larger studies. In addition, the low evidence level of this case report limits the significance considerably, and it remains to be seen whether it is not a purely indeterminate finding. Nevertheless, this case report appears to be a landmark and is therefore important to be reported.

## Data availability statement

The raw data supporting the conclusions of this article will be made available by the corresponding author, without undue reservation.

## Ethics statement

The study involving human participants was reviewed and approved by the Ethics Committee of the University Medical Center Göttingen. Written informed consent for participation was not required for this study in accordance with the national legislation and the institutional requirements. Written informed consent was obtained from the individual(s) for the publication of any potentially identifiable images or data included in this article.

## Author contributions

NH wrote the manuscript. All authors have read, edited, and agreed to the submitted version of the manuscript.
